# Antibody response of definitive hosts against antigens of two life stages of the neuropathogenic schistosome *Trichobilharzia regenti*

**DOI:** 10.1186/s13071-015-1007-y

**Published:** 2015-07-28

**Authors:** Libuše Turjanicová, Libor Mikeš, Monika Pecková, Petr Horák

**Affiliations:** Department of Parasitology, Faculty of Science, Charles University in Prague, Viničná 7, 12844 Prague 2, Czech Republic; Institute of Applied Mathematics and Information Technologies, Faculty of Science, Charles University in Prague, Albertov 6, 128 43 Prague 2, Czech Republic

**Keywords:** Bird schistosome, Trematode, *Trichobilharzia regenti*, avian immunity, IgM, IgY, ELISA, Western blot, Duck, Mallard, *Anas platyrhynchos*

## Abstract

**Background:**

The nasal avian schistosome *Trichobilharzia regenti* spends part of its intravertebrate period of life within the central nervous system. Migration of the parasites can be accompanied by neuromotor disorders or paralysis in natural definitive hosts (ducks) and even in laboratory mammals. Cercariae are also able to penetrate human skin and induce cercarial dermatitis. While the cellular and antibody responses against cercariae and migrating schistosomula have been investigated in mice, little is known about immune reactions in birds. This study first describes the dynamics of antibody response in infected ducks and identifies frequently recognized antigens that may serve as diagnostic markers of infection by *T. regenti*.

**Methods:**

Groups of 35 domestic ducks and 10 mallards were exposed to different doses of *T. regenti* cercariae. Sera were collected at predefined time intervals and tested by ELISA for the presence of specific anti-cercarial IgY and IgM. Antigens recognized by the antibodies were identified on Western blots of cercariae and schistosomula. The applicability in immunodiagnostics was statistically evaluated by expression of specificity and sensitivity values for individual antigens.

**Results:**

In ELISA, the levels of anti-cercarial IgM peaked on day 15 pi. Increased production of IgY associated with the later phases of infection was observed in most individuals around 20 dpi and culminated 30 dpi. The time course of antibody response did not differ among experimental groups, variations were only observed in the levels of specific IgY which depended rather on the age of ducks at the time of infection than on the infectious dose. On Western blots, 40 cercarial and 7 schistosomular antigens were recognized by IgY from infected ducks. Among them, 4 cercarial antigens of 50, 47, 32 and 19 kDa provided the most sensitive and specific reactions.

**Conclusions:**

Antigens of cercariae and schistosomula elicited distinct antibody response in ducks, which correlated positively with the age of animals at the time of infection. Several antigens originating in cercariae and fewer in schistosomula were recognized by IgY with diverse sensitivity and specificity; only a few seemed to be common to both stages. Four of them were considered as the most promising candidates for immunodiagnostics.

**Electronic supplementary material:**

The online version of this article (doi:10.1186/s13071-015-1007-y) contains supplementary material, which is available to authorized users.

## Background

The avian schistosome *Trichobilharzia regenti* Horák, Kolářová et Dvořák, 1998 is a neurotropic nasal parasite of waterfowl, especially ducks. Although birds serve as suitable definitive hosts, cercariae of *T. regenti* have been identified as the aetiological agent of cercarial dermatitis in man [1]. Experimental infections of mice showed that cercariae readily penetrate also mammalian skin and transform to the subsequent stage, schistosomulum, which is able to persist in mice for several days. Schistosomula of the species migrate through peripheral nerves to the spinal cord and brain of both bird and mammalian hosts, and feed on the nervous tissue; damage to the central nervous system (CNS) can give rise to various neuromotor disorders [2–4]. In mammals, however, *T. regenti* is unable to mature and complete its life cycle [2, 5].

Repeated infections of mammals including man lead to an inflammatory reaction known as cercarial dermatitis, which develops after destruction of cercariae in the skin. In sensitive individuals, intensive itching may be accompanied by fever and local lymph node swelling [6–8].

Antibody response of bird hosts against avian schistosome antigens has not yet been studied in detail. Just some antigenic structures recognized by antibodies from infected ducks or mice were shown by immunohistochemistry. In particular, cercarial penetration glands as well as glycocalyx of *T. regenti* cercariae contained antigens triggering immune response. The latter reacted also with antibodies from human patients with known history of cercarial dermatitis and from mice experimentally infected with *T. regenti*; a cross-reaction of antibodies has also been observed with heterologous antigens of the related species *T. szidati* and the human blood fluke *Schistosoma mansoni* [9, 10]. The sera of compatible hosts recognised schistosomular and adult gut associated antigens [10]. Antibodies of the classes IgG and IgE from sera of repeatedly infected mice and patients with confirmed cercarial dermatitis recognized 25 kDa and 34 kDa protein bands on Western blots of *T. regenti* cercarial homogenates [11]. These antigens were identified by mass spectrometry as triose-phosphate isomerase and glyceraldehyde-3-phosphate dehydrogenase [12]. Indeed, host antibody response is also directed towards other developmental stages that are in contact with the host. In addition to the antigens in glycocalyx, penetration glands and tegument of cercariae, also the tegument of schistosomula and adults is recognized by antibodies from infected mice [9].

Here we show the antibody response during the infection by *T. regenti* in specific hosts (ducks). Bird humoral immunity has some specifics compared to mammals. Since the divergence of birds and mammals ca. 300 million years ago, some differences in the antibody responses of those two vertebrate groups have evolved [13]. The most significant departures from mammals include a partly different set of antibody classes, lower variability of Ig binding sites and maturation of B lymphocytes in a specialized immune organ, bursa of Fabricius. Also, mammals generate new antigen-binding sites throughout their life; in contrast, bird antibody diversity is generated only during a brief period of the embryonic development [14].

There are 3 classes of immunoglobulins in birds: IgA, IgM and IgY. Avian IgA has a similar function and structure as in mammals - it is predominant in body secretions and participates in mucosal immunity. IgM is the first class of immunoglobulins being expressed during the embryonic development. It is the predominant isotype produced after initial exposure to a new antigen in primary antibody response. Production of IgY is stimulated in subsequent stages of infection [15]. The IgY molecule is an evolutionary precursor of mammalian antibody classes IgG and IgE [16, 17]. There are two isoforms of IgY in ducks: complete IgY and a smaller version of IgY, called IgYΔFc, which lacks the Fc part of the heavy chain. IgYΔFc is produced during the alternate splicing of mRNA of the heavy chain [18]. This truncated form cannot mediate effector cell functions, therefore it is not very clear why it is produced - maybe it can participate in agglutination of antigenic particles [13].

We aimed to describe the dynamics of antibody response of experimentally infected domestic ducks and wild mallards *Anas platyrhynchos* to various antigens of *Trichobilharzia regenti*, and to identify particular antigens with diagnostic potential. So far, diagnosis at necropsy of infections by schistosomes in birds has been the method of choice. In living hosts, the adult worms of *T. regenti* lay the eggs in the nasal mucosa of birds and, therefore, no eggs are released with faeces to enable coprological examination [8, 19]. As a consequence, immunological methods seem to be a useful alternative in immunoecological studies concerning the influence of pathogens on birds. In addition to the infections of birds, highly antigenic proteins (subsequently produced in a recombinant form) may potentially be used for confirmation of cercarial dermatitis in human patients.

## Methods

### Ethics statement

The maintenance and care of experimental animals was carried out in accordance with the European Directive 2010/63/EU and Czech law (246/1992 and 359/2012) for biomedical research involving animals. Experiments have been performed under legal consent of the Expert Commission of the Section of Biology, Faculty of Science, Charles University in Prague and the Ministry of Education, Youth and Sports of the Czech Republic (ref. no. MSMT-31114/2013-9).

### Experimental animals and infection

One-day-old American Pekin ducklings Cherry Valley strain (*Anas platyrhynchos* f. domestica) have been supplied by Perena Ltd., Chlumec nad Cidlinou (Czech Rep.). Parental animals (breeder ducks) have been vaccinated against viral hepatitis DHAV-1, *Salmonella typhimurium* and *Riemerella anatipestifer*. Ducklings were kept in the animal facility in cages under lamps producing radiant heat – first week at 28 °C, second week at 25 °C, third week at 23 °C and onwards at 18–20 °C. Infection by cercariae of *T. regenti* was performed between 5th and 10th day of life.

Ducklings of wild mallards *Anas platyrhynchos* were obtained at the age of 14 days from the Duck farm Myslív, Klatovské rybářství Plc. (Czech Rep.). At the farm, ducklings have been kept in a heated hall on straw bedding, without contact with external environment. Breeder mallards have been vaccinated against Newcastle disease, viral hepatitis DHAV-1 and duck herpesviral enteritis. Upon arrival to the animal facility, they were kept in a pen at 20 °C. The animals were 22 days old at the time of first infection followed by repeated infections, or 40 days in the case of single infections (the age when serum levels of IgM and IgG reach values similar to those in adult animals) [20, 21].

The life cycle of *Trichobilharzia regenti* has been maintained in the laboratory using snails *Radix lagotis* and domestic ducks as intermediate and final hosts, respectively [22]. Infections of experimental domestic ducks and mallards by cercariae freshly emerged from snails were performed via webs on both feet [23]. The exposure dose was 1500 cercariae per individual. For a single exposure dose, 24 domestic ducks (group SD) and 4 mallards (group SM) were used. As for the repeated infections, 6 domestic ducks (group RD) and 6 mallards (group RM) were used - reinfection was performed with the same dose of 1500 cercariae on days 10, 20, 30 after the initial infection. In a group simulating weak infections expected under natural circumstances, the dose for 5 domestic ducks was 100 cercariae per animal (group LD). In total, 35 domestic ducks and 10 wild mallards were used for experimental infections, and 14 domestic ducks and 3 mallards served as negative non-infected controls.

### Collection of sera

Duck sera were obtained after collection of peripheral blood from *aa. metatarsales dorsales.* Samples were collected in predefined intervals, i.e. 10, 20, 30 and 40 days post-infection for groups RM, SM, RD and first ten animals from the group SD). To refine the picture of the early onset of IgM response, sampling intervals needed to get shortened. Therefore in the case of the other 14 animals from group SD and the whole group LD, blood was collected before infection (day 0) and on days 2, 4, 6, 8, 10, 15, 20, 30 and 40 days post-infection. Aliquots of sera were stored at −20 °C until used.

### Parasite antigen preparation

The infected snails were placed in 100 ml beakers filled with tap water; cercariae of *T. regenti* emerged after illumination for 1 h. Suspensions of cercariae were cooled to 0 °C, concentrated by centrifugation at 1600 × g, washed once with cold water and finally re-suspended in a minimal volume of cold 10 mM phosphate buffer pH 7.2 containing one tablet per 10 ml of a mixture of protease inhibitors (Complete Mini EDTA-free, Roche). Homogenates from cercariae (TrHc) were prepared by 3 cycles of sonication (10 W, 30s) and centrifugation at 16 000 g for 20 min. Supernatants were collected and protein concentration was measured using Quant-iT Protein Assay Kit (Invitrogen). Samples were used immediately or stored at −20 °C.

Schistosomula of *T. regenti* were obtained from spinal cords of domestic ducks dissected 7 days post-infection. Parasites were washed in PBS pH 7.2 several times and the homogenate (TrHs) was prepared as mentioned above.

### ELISA

Due to the limited amount of homogenate from schistosomula, only cercarial antigens were tested by ELISA. TrHc was diluted in carbonate coating buffer (15 mM Na_2_CO_3_ and 35 mM NaHCO_3_, pH 9.6) to protein concentration of 6.25 μg /ml. Flat bottom microtitration ELISA plates (MaxiSorp, Nunc) were coated at 4 °C overnight (100 μl/well). Blocking was performed with 2 % casein (Sigma) in PBS containing 0.05 % Tween20 (PBS-T) for 2 h. Wells were incubated with duck sera diluted 1:160 in PBS-T for 2 h, washed 3 × 1 min with 150 μl of PBS-T and then incubated with HRP-labelled goat anti-duck “IgG” (= anti-duck IgY, whole molecule, supplied under this “erroneous label” by KPL) diluted 1:500 in PBS-T or anti-IgM (Nordic Immunology) diluted 1:5000 in PBS-T (according to the manufacturer’s recommendations) for 1.5 h. The wells were washed 6 × 1 min with 150 μl of PBS-T and developed using tetramethylbenzidine (TMB) substrate kit (Sigma T0440). Reaction was stopped by addition of 1 M HCl (100 μl/well) and read at 450 nm using Infinite M200 reader (Tecan, Austria). The lower level detection cut-off for each time point was determined [24].

### Western blotting

Homogenates of cercariae or schistosomula (TrHc or TrHs) were separated individually on 4-12 % gradient NuPAGE® Bis-Tris precast mini IPG gels (Invitrogen). The load was 250 μg protein/IPG well for TrHc and 200 μg protein/IPG well for TrHs. Precision Plus Protein Dual Xtra standards (Bio-Rad) were used. Proteins were transferred onto Immuno-Blot PVDF membranes (Invitrogen) using Trans-Blot Turbo Transfer System (Bio-Rad). The membrane was cut into stripes and blocked with 5 % non-fat milk (Bio-Rad) in PBS-T. Membrane stripes were incubated individually with duck sera diluted 1:100 in PBS -T at RT for 2 h. After washing 3 × 5 min in PBS-T, secondary antibodies were applied - 1:1000 HRP-labelled goat anti-duck IgG (KPL) and/or 1:5000 anti-IgM (Nordic Immunology) in PBS-T. Following final wash 3 × 5 min in PBS-T, blots were developed using Opti-4CN Substrate Kit (Bio-Rad). Developed membranes were scanned using GS-800 densitometer (Bio-Rad). In total, 186 domestic duck and mallard serum samples from all experimental groups collected in various time points post-infection and 68 sera from control collections were tested with cercarial antigens (TrHc). Collection of schistosomula from infected ducks is difficult and requires euthanasia of experimental animals, therefore the amount of antigen and subsequently the number of sera tested with homogenate of schistosomula (TrHs) were limited. Altogether, we tested 29 domestic duck and mallard positive serum samples - 20 sera from 6 domestic ducks group SD, 1 serum group RD, 6 sera from 2 domestic ducks group LD, 1 serum group RM and 1 serum group SM, representing all time slots post-infection, and 6 sera from control animals.

### Statistical analysis

**Fig. 1:**
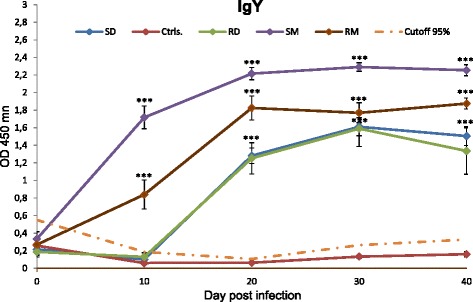
The course of specific IgY response against *T. regenti* cercarial antigens in ELISA. SD - domestic ducks exposed to single dose of 1500 of cercariae; RD - domestic ducks exposed repeatedly to 1500 cercariae; SM - mallards infected by single dose of 1500 cercariae at the age of maturity of antibody response; RM - mallards exposed repeatedly to 1500 cercariae; Ctrls - control non-infected animals. Cutoff calculated for 95 % confidence level is drawn by a dot-and-dash line for each time point. Data are the mean ± SEM for at least five individuals. Statistical significances (***P < 0.001) were confirmed by random effects ANOVA

**Fig. 2:**
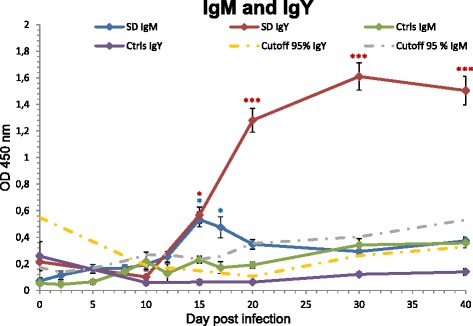
Comparison of the course of specific IgM and IgY responses against *T. regenti* cercarial antigens in ELISA. SD - domestic ducks exposed to single dose of 1500 cercariae; Ctrls - control non-infected ducks. Cutoff calculated for 95 % confidence level is drawn by a dot-and-dash line for each time point. Data are the mean ± SEM for at least five animals. Statistical significances (*P < 0.05; ***P < 0.001) were confirmed by random effects ANOVA

Statistical analyses of reactions of duck antibodies with antigens in ELISA were performed using random effects ANOVA. Data set of OD presented in Fig. 1 was not transformed, while the data presented in Fig. 2 were transformed by ln(OD + 1). Data values with P < 0.001, P < 0.01, and P < 0.05 were considered significant. All data sets were tested for normality (Q-Q plot) and for equality of variances (two-variances F-test). Results presented in graphs are mean values with standard errors of the mean of at least five samples per group.

Molecular sizes of antigens on Western blots were measured from scanned membranes by Quantity One software (Bio-Rad). Sizes of individual identical antigens were averaged after careful inspection of the putative identities. Standard error of the mean was determined for each individual antigen. In graphs and table (supplementary data), only antigens recognized by at least 10 % of the tested sera are presented for particular time slots. For each individual cercarial antigen, the values of specificity and sensitivity were calculated.

## Results

### Dynamics of specific IgY and IgM responses against *T. regenti* cercarial antigens in ELISA

In infected domestic ducks and mallards, both a single dose and repeated infections by *T. regenti* cercariae (groups SD, RD, LD, RM, SM) caused significant increase in levels of specific anti-TrHc antibodies of the IgY class. The statistical significance was confirmed by random effects ANOVA: groups SD 10 dpi and RD 10 dpi did not exceed the level of significance; for all other time slots and groups P < 0.001. The following results (except for group LD) are graphically summarized in Fig. 1.

The level of anti-TrHc IgY antibodies increased between day 0 and day 10 pi. in the case of both experimental groups of mallards (SM and RM). For the groups of domestic ducks SD, RD and LD, significant increase of OD was observed between days 10 and 20 pi and the levels of specific IgY peaked 30 days post-infection. Thereafter they started to decrease slightly over the time course.

In group SM, the shape of the curve of anti-TrHc IgY response was similar as for the group SD. The main difference between one-time infected young domestic ducks and older mallards concerned the value of OD, which was approx. 1.4 times higher for the latter group in the peak of IgY production.

In the groups of repeatedly infected mallards and domestic ducks (RM and RD), the course of specific anti-TrHc IgY response resembled the situation in animals infected only once. Since day 20 pi, the levels of specific IgY in mallards were stable till the end of the experiment. The values of OD were similar for repeatedly and one-time infected animals at corresponding time points.

The dynamics of production of specific anti-TrHc IgY antibodies in the group of domestic ducks exposed to a low dose of 100 cercariae (LD) was similar to that in domestic ducks exposed to 1500 larvae (not shown).

A graphical summary of the course of specific IgM response and comparison with the dynamics of IgY response in one-time infected domestic ducks can be found in Fig. 2. The levels of IgM in sera of the group SD started to rise slightly as early as 2 dpi. A more rapid increase in specific IgM levels could be observed after 10 dpi, with a peak on day 15 pi, then followed by a gradual decrease. The statistical significance was confirmed by random effects ANOVA: for group IgM 15 dpi and 17 dpi P < 0.05. All other time slots were under the level of significance.

### Western blot detection of *T. regenti* cercarial antigens

**Fig. 3:**
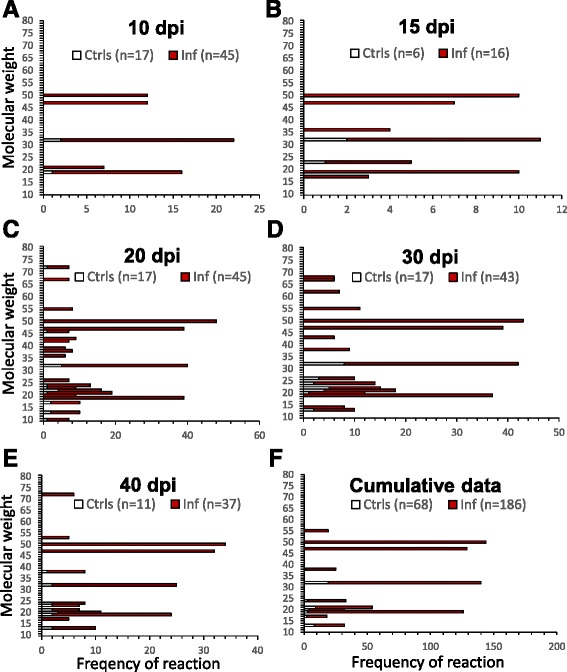
Cercarial antigens recognized on Western blots by IgY from domestic ducks and mallards of all experimental groups. Antigens are ordered according to their molecular weights (axis Y). Data are expressed as the numbers of sera (axis X) recognizing particular antigens. Red bars (Inf) - sera from infected animals; white bars (Ctrls) - sera from control non-infected animals. Charts A-E correspond to the time slots post-infection, chart F contains cumulative data from all time slots. Numbers of tested sera from particular groups of infected domestic ducks and mallards are in parentheses: **a** - 10 dpi (24 SD, 6 RD, 6 RM, 5 LD, 4 SM); **b** - 15 dpi (11 SD, 5 LD); **c** - 20 dpi (24 SD, 6 RD, 6 RM, 5 LD, 4 SM); **d** - 30 dpi (23 SD, 5 RD, 6 RM, 5 LD, 4 SM); **e** - 40 dpi (18 SD, 5 RD, 5 RM, 5 LD, 4 SM); **f** - cumulative results from all time intervals. Antigens reacting with less than 10 % of sera were omitted. Only antigens in the range between 10 and 80 kDa are shown for better clarity

**Fig. 4:**
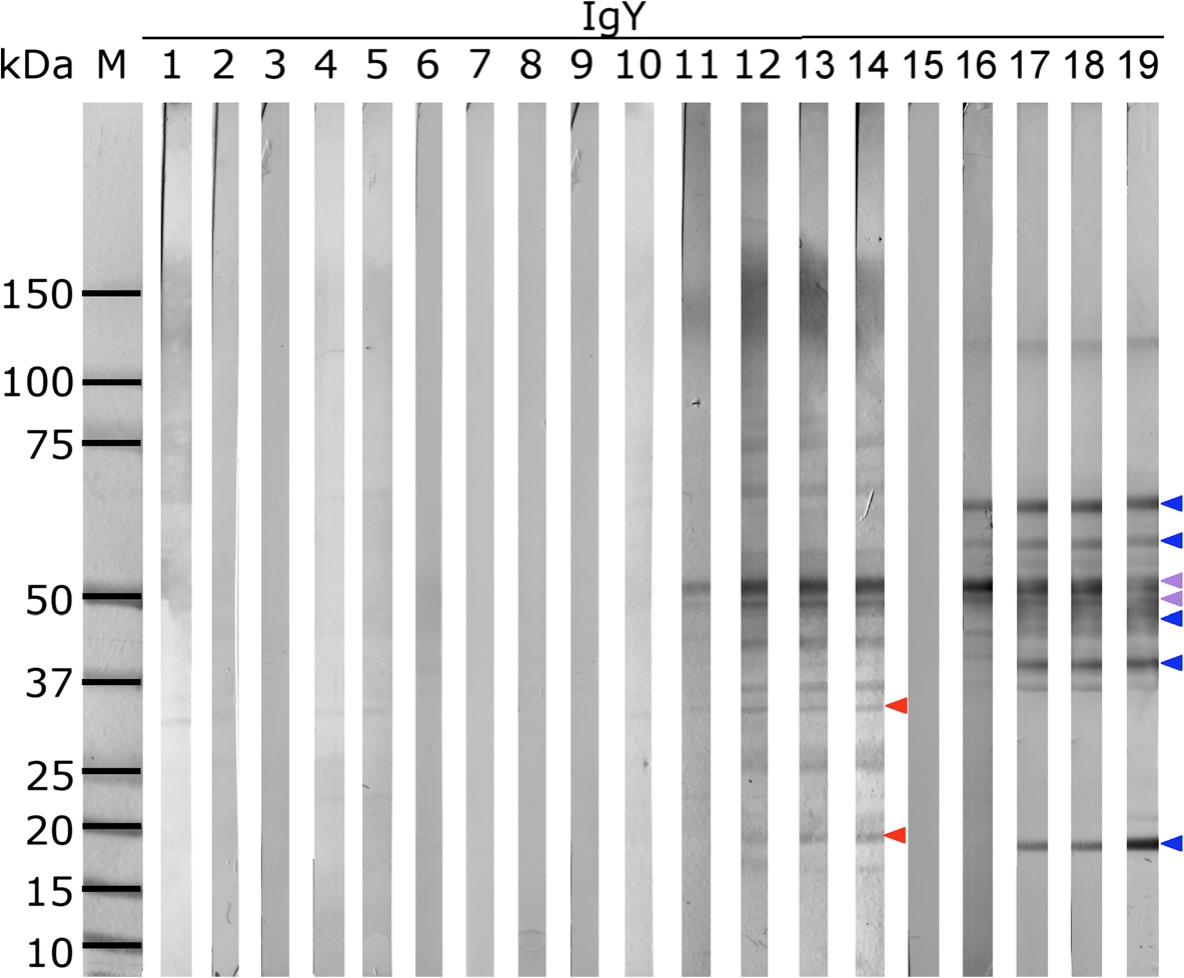
Profiles of antigens recognized by domestic duck IgY in homogenates of cercariae and schistosomula on Western blots. Reactions with sera from domestic ducks exposed to a single dose of 1500 cercariae (group SD) are shown as an illustrative example of the reactions. Cercarial homogenate = lanes 1–5 and 10–14. Homogenate of schistosomula = lanes 6–9 and 15–19. Lane M - markers of MW; lanes 1, 2, 3, 4, 5 - sera of an infected duck taken on days 10, 15, 20, 30 and 40 after initiation of the experiment; lanes 6, 7, 8, 9 - sera of a non-infected duck on days 10, 20, 30 and 40 days; lanes 10, 11, 12, 13 and 14 - sera of an infected duck on days 0, 10, 20, 30 and 40 pi; lanes 15, 16, 17, 18 and 19 - sera of an infected duck on days 0, 10, 20, 30 and 40 pi. Red arrowheads - cercarial antigens 32 and 19 kDa specifically recognized by IgY; blue arrowheads – antigens of schistosomula 64, 54, 44, 39 and 18 kDa specifically recognized by IgY; violet arrowheads - antigens 50 and 47 kDa commonly recognized by IgY in both cercariae and schistosomula

**Fig. 5:**
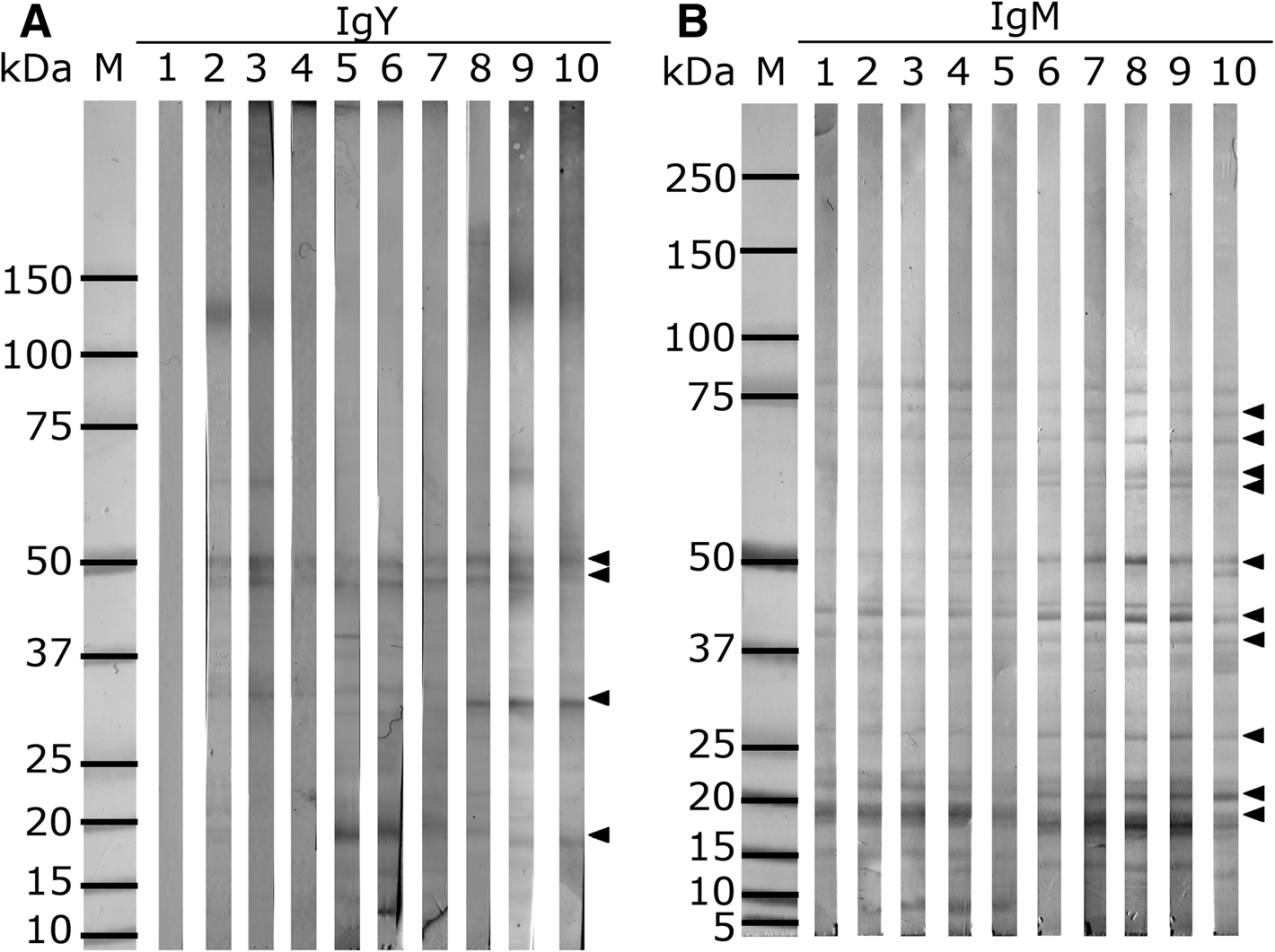
Profiles of cercarial antigens recognized by domestic duck and mallard IgY and domestic duck IgM on Western blots. Only illustrative examples are shown. Lanes M - markers of MW. **a** - IgY response. Lane 1 - serum of a control domestic duck (group RD) 40 days after initiation of the experiment; lanes 2, 3, 4 - sera from a repeatedly infected domestic duck (group RD) 10, 20 and 30 days after first infection; lanes 5, 6 and 7 - serum of a domestic duck exposed to low dose of 100 cercariae (group LD) 20, 30 and 40 dpi; lane 8, 9 and 10 - sera of a “mature” mallard exposed once to 1500 cercariae (group SM) 20, 30 and 40 dpi. Arrowheads mark the proteins of 50, 47, 32 and 19 kDa that are specifically recognized by IgY. **b** - IgM response of domestic ducks exposed once to 1500 cercariae (group SD). Lanes 1, 2, 3, 4 and 5 - sera from a control duck 0, 10, 15, 20, 30 and 40 days after initiation of the experiment; lane 6 - serum of an experimental duck before infection; lanes 7, 8, 9 and 10 - sera of an infected duck 10, 15, 20, 30 and 40 dpi. Arrowheads point to the proteins of 75, 71, 50, 40, 37, 26, 20 and 17 kDa reacting with IgM from both control and infected ducks, and to the antigens of 64 and 62 kDa which were recognized specifically by IgM from the infected ducks only

Using 186 domestic duck and mallard serum samples from all experimental groups collected in various time points post-infection and 68 sera from control collections, we found around 40 antigens recognized by IgY class antibodies from infected animals. Western blot profiles showed protein bands between 10 and 140 kDa. Antigens which were recognized by at least 10 % of the tested sera taken at particular time slots from infected individuals, and their sensitivity/specificity values are summarized in the table in Additional file 1. Graphical display of the results is shown in Fig. 3. Reactivity of IgY with cercarial antigens was followed at time points of 10 dpi (Fig. 3a), 15 dpi (Fig. 3b), 20 dpi (Fig. 3c), 30 dpi (Fig. 3d) and 40 dpi (Fig. 3e). In Fig. 3f there is an overall expression of the results, showing cumulative data on the reactivity of sera without regard to the time of collection (again, the antigens reacting with less than 10 % sera were omitted).We noticed 4 frequently recognized antigens with molecular weights of 50 kDa (Tr50), 47 kDa (Tr47), 32 kDa (Tr32) and 19 kDa (Tr19) (Figs. 3f, 4 and 5a), among which the antigens Tr32 and Tr19 were occasionally nonspecifically recognized also by control sera from non-infected animals. The number of recognized antigens as well as density of bands on blots increased with time post-infection till day 30 pi (Figs. 3a-d, 4 and 5a).

Binding of IgM class antibodies to cercarial antigens of molecular weights 75, 71, 50, 40, 37, 26, 20 and 17 kDa was observed for sera from infected animals as well as for sera from the control non-infected group. In the case of infected individuals, these antigens displayed more dense bands given by stronger colorimetric reaction. We noticed specific reactions with antigens of molecular weights 64 and 62 kDa, but only in some infected animals (Fig. 5b).

### Western blot detection of antigens from schistosomula of *T. regenti*

**Fig. 6:**
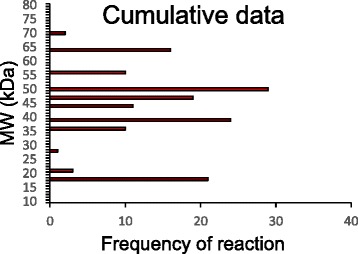
Antigens of schistosomula recognized on Western blots by IgY from infected domestic ducks and mallards. Complete cumulative data (20 SD, 1 RD, 6 LD, 1 RM and 1 SM) for reactions of IgY from selected sera of all experimental groups of domestic ducks and mallards at various time points post-infection are shown. Antigens are ordered according to their molecular weights (axis Y). Data are expressed as the numbers of sera recognizing particular antigens (axis X). Red bars - sera from infected animals. No reaction of control sera was observed. Only antigens in the range between 10 and 80 kDa are shown for better clarity

The reactivity of sera from infected ducks with antigens of the post-cercarial developmental stage – schistosomulum – was also investigated. Altogether, we used 29 domestic duck and mallard serum samples collected at various time points post-infection, and 6 sera from control animals. Among all reacting antigens, those of molecular weights 64, 54, 50, 47, 44, 39 and 18 kDa were recognized significantly by sera from different infected individuals (Fig. 6). Comparison of the spectra of antigens from homogenates of cercariae and schistosomula recognized specifically by IgY showed that although the patterns differed noticeably between the two life stages, two bands of the same sizes occurred in both of them - Tr47 and Tr50 (Figs. 4 and 6).

## Discussion

Our study describes the development of antibody responses of natural definitive hosts against antigens of the bird schistosome *T. regenti*. The major part of the experiments was performed with a domestic strain of ducks. For comparison, in order to include more genetic variability of the hosts which could affect antibody response, a smaller number of wild mallards was used. The results of ELISA showed that the course of IgY response against the antigens of cercariae is similar between domestic ducks and mallards, but in mallards there was a quicker onset and earlier peak of IgY response, and the levels of antibodies were higher. This could be caused by the age of mallards at the time of infection; especially in the group of mallards infected at the age of 40 days (group SM) the immune system reached almost its maturity [20, 21]. Nevertheless, the results showed that significant levels of specific antibodies can be detected even in ducklings infected at a low age – this means that immunodiagnostics of *Trichobilharzia* infections is possible even in very young birds which may be infected more frequently than adults [25].

The higher level of IgY depended rather on the age of mallards/domestic ducks than on the antigenic boosting due to repeated infections by cercariae; this is also clearly evidenced in the case of single and repeated infections of domestic ducks (groups SD and RD) which were of equal age, and challenge infections did not elicit a higher level of IgY (Fig. 1). Surprisingly, low exposure doses by 100 cercariae produced roughly the same IgY levels as regular exposures to 1500 cercariae, suggesting that immunodiagnostics of *T. regenti* infections may be useful under natural conditions where the birds are probably invaded by low numbers of larvae. High levels of specific IgY remained in duck sera 40 days pi when the experiments were terminated; at this time the infection by *T. regenti* was already inactive and parasitological dissections did not detect adult worms or eggs in the nasal mucosa [2, 26]. Furthermore, pathological changes typical for *T. regenti* infections could be observed in the nasal mucosa [26]. In mice infected by *T. regenti* the response against cercarial antigens increased rapidly after the first infection and stayed at a relatively high level till day 120 post-infection [11]. As it is evident that *T. regenti* is a short-living schistosome, immunodiagnostics seems to be a good tool for uncovering the history of infections by this species in both natural and accidental hosts. Unfortunately, the conditions of our animal facility did not allow keeping of experimental ducks for a longer time without circumvention of law, therefore steadiness/fading away of the antibody levels could not be mapped further on.

Monitoring by ELISA of the dynamics of specific IgM raised against cercarial antigens showed a gradual increase and a significant peak 15 dpi. The IgM response is short lived. The highest level of IgM can be recorded in the period corresponding to 4–8 days after inoculation of antigen and then declines rapidly [15]. However, this assumption relates to adult birds. The delayed production in our case could be due to the immaturity of the immune system in young experimental animals. In the group of young domestic ducks, we observed first increase 5 dpi, which is in accordance with [27]. Indeed, the results of various studies mentioned above are hardly comparable because of the application of different antigens/pathogens.

On Western blots of cercarial homogenates, we found around 40 antigens recognized by IgY from infected domestic ducks and mallards. Some were recognized only seldomly; to keep the evaluation of data uncluttered, we omitted presentation of those antigens which were recognized by less than 10 % of sera and have therefore been considered irrelevant for immunodiagnostics. Among the 29 antigens recognized to a higher extent (Fig. 3 and Additional file 1), some were tagged also by IgY from animals which have not encountered infection by bird schistosomes. These were considered not enough specific for diagnostic purposes. In fact, many other antigens have been recognized by sera with specificity above 90 %, but the overall sensitivity was always below 25 % and often less than 10 %. The most specific and sensitive reactions include four proteins provisionally termed Tr50, Tr47, Tr32 and Tr19, among which the first two were recognized by IgY only from the infected individuals, thus being 100 % specific. The other two showed occasional reactions with control sera (lower specificity) - ca. 72 % and 96 % for Tr32 and Tr19, respectively. The four antigens reacted even with some sera taken in early phases of infections (sensitivity 27 – 44 % on day 10 pi – Fig. 3a, Additional file 1) and all experimental groups of domestic ducks and mallards, and, indeed, dominated among the other recognized antigens after evaluation of cumulative data (Fig. 3f, Additional file 1). Overall sensitivity reached values between ca. 65 – 77 %; the highest was for Tr50 (77 %) in the case of cumulative data. This was given by somewhat lower number of sera reacting with these antigens in early phases of infections, but on day 30 pi the sensitivity was 100 % for Tr50, 91 % for Tr47 and 79 % and 86 % for Tr32 and Tr19. The values of sensitivity for these antigens dropped towards day 40 pi (more rapidly for Tr32 and Tr19), suggesting that specific IgY in some infected animals started to disappear. This seems to be in accordance with the results of ELISA where the peak or IgY responses occurred around day 30 pi.

In the time course of infection, the number of antigens recognized by IgY from more than 10 % of tested sera has been increasing; in this regard, a peak occurred 20 dpi, when 22 antigens were detected on blots. On day 30 pi there were 17 antigens tagged and the number further dropped on day 40 pi (Fig. 3). Comparison of blots with cercarial homogenates reacting with sera from various experimental groups showed intra-group variations, but no substantial differences in variability among particular groups of domestic ducks and mallards. The outbred ducks used in our experiments can react more individually depending on their genetic background than the inbred animals which were not available for our study. This can explain the observed differences in recognition of some antigens of the parasite [28]. Nevertheless, the four most promising antigens mentioned above in terms of immunodiagnostics of infections by *T. regenti* in definitive hosts were recognized by animals infected with either high or low dose of cercariae, one-time or repeatedly.

The exact identification of antigens (e.g., by mass spectrometry) inducing IgY response in ducks has not yet been performed, so we can only assume that Tr50 discovered in our study could be identical to a 50 kDa protein detected on blots of cercarial antigens with IgG from mice repeatedly infected by *T. regenti* [11]. Moreover, in penetration glands of cercariae, a 32 kDa cysteine peptidase cathepsin B2 was found [29, 30] which is released during penetration of cercariae through the host skin. Thus, Tr32 might be this enzyme.

The analysis of IgM response against cercarial antigens on Western blots revealed numerous bands which reacted not only with the sera from infected individuals, but also with controls. Thus, the specificity was low in most cases. In addition to 8 antigens specifically recognized by immune sera at least in some cases, 2 antigens (64 and 62 kDa) were considered to be recognized specifically, but with rather low sensitivity. This made the possibility of immunodetection by IgM in the early phases of infection doubtful.

As schistosomula can be obtained from the CNS of infected animals only at necropsy, we tested on blots only a limited number of sera with this kind of antigen – therefore the specificity/sensitivity values were not calculated. Few antigens were identified (64, 54, 50, 47, 44, 39 and 18 kDa) that were specifically recognized on a larger scale by IgY from different groups of infected animals at various time slots post-infection. The total number of recognized antigens was lower in comparison with the homogenate of cercariae. This is consistent with the results of a previous study [9] in which the immunoreactivity of histological sections of schistosomula and adult worms of *T. regenti* with sera of infected hosts was lower comparing to cercariae. We found only two sensitive antigens recognized by IgY that had the same molecular size (50 and 47 kDa) in the protein profiles from cercariae and schistosomula. This was surprising as the data from ongoing transcriptomic analysis revealed that around 87 % of transcripts are common to both stages (unpublished data). High immunogenicity of these two antigens in cercariae and schistosomula could be responsible for relatively high levels of specific IgY even 40 dpi, when the parasite is already not present in the host.

## Conclusions

This study showed that immunodiagnostics of infections by *T. regenti* in specific definitive hosts is possible, namely on the basis of IgY response. Both ELISA and Western blotting seem to be applicable methods. The latter showed that there are few antigens in cercariae and schistosomula which are recognized with excellent specificity and relatively high sensitivity. We also brought the evidence that a significant antibody response is directed against the antigens of schistosomula isolated from the CNS. This is of interest as the CNS is often believed to be an immunoprotective compartment in the host body. Therefore, it needs to be studied further if the antigens expressed by the intra-CNS schistosomula stimulate the antibody response in a direct way. Characterization of the molecular nature of these antigens is in progress. Once produced as recombinant proteins and proved as reliable targets for specific antibodies, they could provide a robust tool for diagnostics of *T. regenti* infections in natural bird hosts, and possibly also in accidental mammalian hosts including human patients with cercarial dermatitis.

## Additional file

Additional file 1:
**Summary of cercarial antigens recognized on Western blots by IgY from all groups of domestic ducks and mallards at particular time points.** Antigens are ordered according to their mean MW, standard errors of the means are shown in parentheses (first column). Particular time points of collection of sera from either control (Ctrls) or infected (Inf) animals together with the number of sera tested are shown in the first row (dpi - days post-infection). Data in cells represent: the number of sera which reacted with a particular antigen/expression of specificity (and/or sensitivity) in per cent. Cumulative data for control and infected animals are shown in the last two columns. Only antigens reacting with at least 10 % of the tested sera samples from at least one particular time point are included. Highlighted in yellow are the four most sensitive antigens Tr50, Tr47, Tr32 and Tr19.
